# A reporter mouse model for *in vivo* tracing and *in vitro* molecular studies of melanocytic lineage cells and their diseases

**DOI:** 10.1242/bio.025833

**Published:** 2017-06-22

**Authors:** Melissa Crawford, Valerie Leclerc, Lina Dagnino

**Affiliations:** Dept. of Physiology and Pharmacology, Children's Health Research Institute and Lawson Health Research Institute, The University of Western Ontario, London, Ontario N6A 5C1, Canada

**Keywords:** Melanocytes, Genetically engineered mouse reporter models, Extracellular matrix, Migration, Dendricity

## Abstract

Alterations in melanocytic lineage cells give rise to a plethora of distinct human diseases, including neurocristopathies, cutaneous pigmentation disorders, loss of vision and hearing, and melanoma. Understanding the ontogeny and biology of melanocytic cells, as well as how they interact with their surrounding environment, are key steps in the development of therapies for diseases that involve this cell lineage. Efforts to culture and characterize primary melanocytes from normal or genetically engineered mouse models have at times yielded contrasting observations. This is due, in part, to differences in the conditions used to isolate, purify and culture these cells in individual studies. By breeding ROSA^mT/mG^ and *Tyr::CreER^T2^* mice, we generated animals in which melanocytic lineage cells are identified through expression of green fluorescent protein. We also used defined conditions to systematically investigate the proliferation and migration responses of primary melanocytes on various extracellular matrix (ECM) substrates. Under our culture conditions, mouse melanocytes exhibit doubling times in the range of 10 days, and retain exponential proliferative capacity for 50-60 days. In culture, these melanocytes showed distinct responses to different ECM substrates. Specifically, laminin-332 promoted cell spreading, formation of dendrites, random motility and directional migration. In contrast, low or intermediate concentrations of collagen I promoted adhesion and acquisition of a bipolar morphology, and interfered with melanocyte forward movements. Our systematic evaluation of primary melanocyte responses emphasizes the importance of clearly defining culture conditions for these cells. This, in turn, is essential for the interpretation of melanocyte responses to extracellular cues and to understand the molecular basis of disorders involving the melanocytic cell lineage.

## Abstract (precis)

**Summary:** A reporter mouse to trace melanocytic lineage cells was generated by breeding existing mouse lines. Methods were also developed to characterize the proliferation and motility of primary mouse melanocytes.

## INTRODUCTION

Genetic alterations that alter survival, migration, proliferation or differentiation of embryonic neural crest cells give rise to a variety of human disorders, collectively termed neurocristopathies. The latter include pigment, skin, thyroid and hearing disorders, as well as craniofacial and cardiac abnormalities (Noisa and Raivio, 2014). Mutations in several genes implicated in neural crest cell function are responsible for various human developmental defects, such as Waardenburg syndrome, characterized by sensorineural hearing loss and pigmentation anomalies in hair, skin and irises (Baxter et al., 2004; Etchevers et al., 2006). Waardenburg syndrome accounts for about 3% of congenital deafness worldwide (Issa et al., 2017). Significantly, many of the genetic mutations associated with human neurocristopathies also occur in rodents, manifesting themselves in the form of reduced and/or altered pigmentation phenotypes.

In mice, a subset of neural crest cells adopts a melanocytic fate around E9.5-10.5, becoming melanoblasts, which begin to spread from areas adjacent to the neural tube and through the developing dermis. Melanoblasts proliferate, and interact with their microenvironment to localize to specific body sites. They migrate along a dorsolateral path towards the face, ventral abdomen and the developing limbs. In the truncal region, melanoblasts move within the dermis, and begin to penetrate into the epidermis around E11.5. Melanoblasts localize to the epidermis by E15.5 and, by E16.5, they also home in developing hair follicles (Li and Machesky, 2012; Luciani et al., 2011; Mort et al., 2015). Melanoblasts subsequently establish a melanocyte stem cell population, which can self-renew or differentiate into melanin-producing mature melanocytes postnatally (Mort et al., 2015).

Naturally occurring mouse coat color mutants have provided valuable information regarding the pathways that regulate melanocytic cell specification, proliferation, survival, migration and stem cell renewal (Baxter et al., 2004). For example, in heterozygous dominant megacolon mice, white spotting is evident. This phenotype arises from a point mutation in the *Sox10* gene, which introduces a premature stop codon. Sox10 activates *Mitf* transcription, functions during melanocyte specification, and maintains melanocyte-specific gene expression later in development (Herbarth et al., 1998; Wegner, 2005). Genetically engineered mutant mouse models that use Cre recombinase-mediated targeting of genes in pigment-producing cells have also been generated and used to examine the roles of many genes in melanocytic cell specification, homing, maturation and survival. Amongst them are included transgenic lines that express Cre or Cre fused to a modified estrogen receptor (CreER^T2^) under the control of the *Mitf*, *Tyr*, *Dct*, *Tyrp1* or *MART-1* (*Melan-A)* promoter (Alizadeh et al., 2008; Aydin and Beermann, 2011; Bosenberg et al., 2006; Colombo et al., 2007; Delmas et al., 2003; Guyonneau et al., 2002; Mori et al., 2002). Some of these transgenes additionally target non-melanocytic cells; others express Cre at low levels, whereas in tamoxifen-inducible Cre lines, targeting of relatively small fractions of cells is often observed. For example, in *Dct::Cre* mice, melanoblasts and melanocyte stem cells are targeted, as well as cells in the telencephalon (Guyonneau et al., 2002). *Tyrp1::Cre* mice exhibit Cre expression in retinal pigmented epithelium and, ectopically, in the neural retina (Mori et al., 2002), and the efficiency of tamoxifen induction of Cre-mediated recombination can range from 12% to >80% (Bosenberg et al., 2006). The development of mouse genetic tools and imaging approaches that exploit the properties of fluorescent proteins specifically expressed in the melanocytic lineage, and which allow *in vivo* and *ex vivo* studies of melanocytic cell characteristics, as well as molecular studies of melanocytic cell behavior, has been more limited (Colombo et al., 2012; Li et al., 2011; Woodham et al., 2017). Moreover, the use of melanocytic cells cultured under a variety of conditions, together with studies using melanoma tumor cells as surrogates to model normal melanocytic cells, can yield contrasting information and significantly complicate the elucidation of cellular and molecular processes important in these cells. In an effort to address this void, we have bred ROSA^mT/mG^ and *Tyr::CreER^T2^* mice, generating animals in which melanocytic lineage cells are identified through expression of green fluorescent protein. We have also defined the growth characteristics of primary melanocytes isolated from mouse epidermis, and now describe the diverse responses of these cells to various extracellular matrix substrates. Our models will facilitate a more systematic exploration and understanding of melanocytic lineage cell biology, and its alterations in various human pigmentation disorders and neurocristopathies.

## RESULTS

### Generation of a melanocytic lineage reporter mouse model for cell fate mapping

**Fig. 1.**
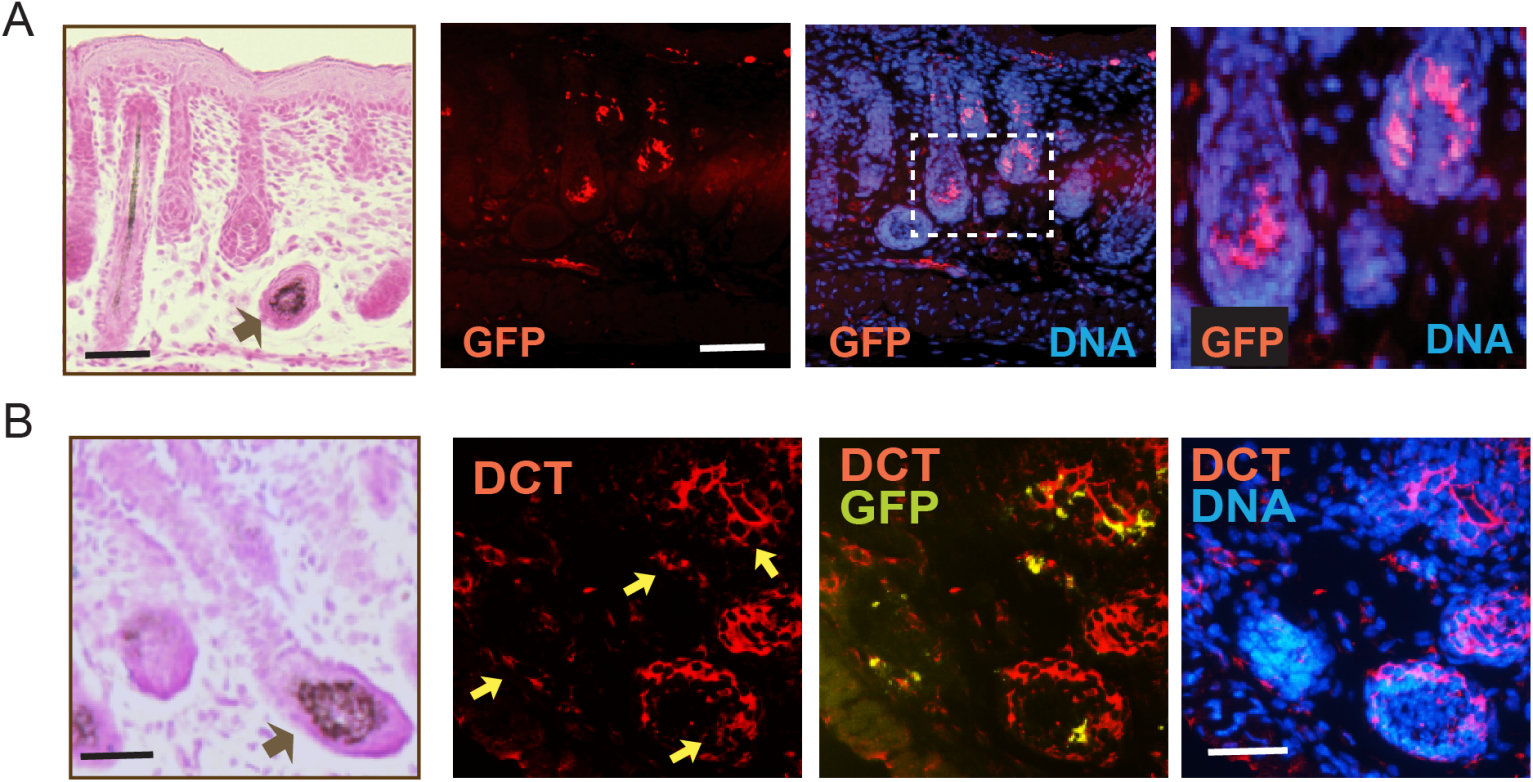
***In vivo* tracing of melanocytic lineage cells.** Skin sections from E20.5 embryos with a *Tyr::CreERT2-mT/mG* background harvested from a dam treated with tamoxifen at 11.5 days of gestation. Tissue sections were processed with Fontana-Masson staining (FM) and visualized with light microscopy to highlight melanin-producing melanocytic cells (indicated by the black arrows). Adjacent skin sections were processed for immunofluorescence microscopy with antibodies against the indicated proteins. DNA was visualized with Hoescht 33342. (A) Sections probed with anti-GFP antibodies show targeted melanocytes in the hair follicle. Inset is shown at higher magnification in the adjacent micrograph. (B) Specimens were probed with anti-GFP and anti-DCT antibodies to demonstrate the melanocytic lineage of GFP-positive cells. Yellow arrows in DCT panel indicate melanocytic cells that also express GFP, as indicated in the DCT-GFP overlay panel. Scale bars: 25 µm.

Melanocytes arise from precursor melanoblasts, which begin to migrate from the mouse neural crest at embryonic day (E)9.5, and by E16.5 are found in both the epidermis and the hair follicles (Luciani et al., 2011; Mort et al., 2015). We have generated a reporter mouse model that allows tracing of melanoblasts and their progeny to better examine the processes involved in melanoblast migration, survival and differentiation into pigment-producing melanocytes. These animals, hereafter termed *Tyr::CreERT2-mT/mG*, have been bred into a reporter *ROSA^mT/mG^* background and express specifically in cutaneous melanocytic cells Cre recombinase fused to a modified estrogen receptor (CreER^T2^) under the control of the *Tyr* promoter, beginning at E10.5 (Bosenberg et al., 2006; Muzumdar et al., 2007). Tamoxifen administration to these mice activates CreER^T2^ and induces expression of a membrane-targeted form of green fluorescent protein (mGFP). Non-targeted cells constitutively express mTomato, a membrane-targeted red fluorescent protein form. Analysis of E20.5 skins harvested from embryos exposed to tamoxifen at E11.5 days revealed mGFP immunoreactivity detected exclusively in a subpopulation of cells in the hair follicle (Fig. 1A) and in the interfollicular epidermis (data not shown). These cells also expressed the melanocytic-specific enzyme dopachrome tautomerase (DCT), confirming the melanocytic origin of the targeted cells (Fig. 1B). No detectable recombination was observed in embryos from dams treated with vehicle only (data not shown).

### Characteristics of cultured melanocytes

**Fig. 2.**
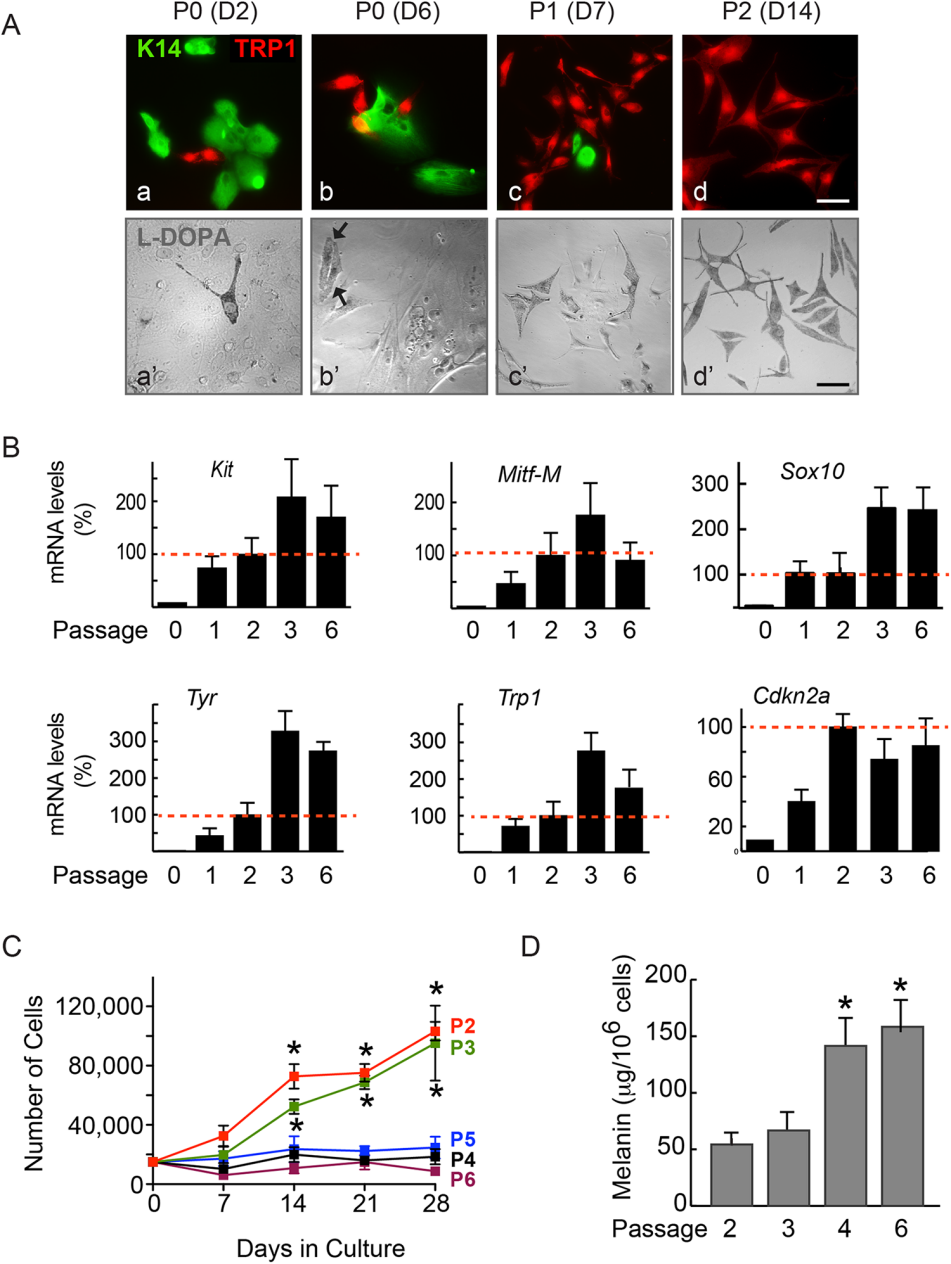
**Characteristics of primary mouse melanocyte cultures.** (A) Epidermal cell suspensions were cultured for the indicated number of days (D) and passages (P). The cells were processed for immunofluorescence microscopy to detect keratin 14 (K14) in keratinocytes and TRP1 in melanocytes (a,b,c,d), or stained with L-DOPA, which is converted to a brown pigment only in melanocytes (a′,b′,c′,d′). Arrows indicate the melanocytes in P0 cultures. Scale bars: 50 µm. (B) mRNA was isolated from melanocyte cultures at the indicated passage numbers, reverse transcribed and analyzed by qPCR to determine the relative abundance of the indicated transcripts. The results were obtained from three technical replicates per cDNA tested, and are expressed as the mean+s.d. from three independent cell isolates, relative to mRNA levels in P2 cells, which are set to 100% (red dotted line), because P2 cells are the earliest passage composed of ≥95% melanocytes. (C) Melanocytes at the indicated passages were cultured, trypsinized and the number of trypan blue-excluding cells was determined at the indicated intervals following seeding. The data were obtained from duplicate samples for each of three independent cell isolates, and are expressed as mean±s.d., and *P<0.05 relative to the number of cells seeded at D0 (*n*=3, two-way ANOVA). (D) Melanin was extracted and quantified in cultures at the indicated passages. The data are expressed as mean melanin content+ s.d., and *P<0.5 relative to melanin in P2 cells (*n*=3, one-way ANOVA).

The isolation of primary melanocytes and their culture to high purity is a prerequisite for thorough cellular and molecular characterization of biological processes in melanocytic lineage cells. We generated single-cell suspensions from newborn *Tyr::CreERT2-mT/mG* mouse epidermis mainly composed of melanocytes and keratinocytes. The cells were cultured in selective growth medium supplemented with endothelin-3 (ET-3), which we found favored melanocyte growth. 48 h after isolation, we observed mixed cultures composed of 9% tyrosinase-related protein 1 (TRP1)-positive melanocytic cells, which increased to 29% after 6 days, as the culture conditions did not allow contaminating keratinocytes to proliferate (Fig. 2A). Through the combination of differential trypsinization (Huang et al., 2013) and serial passaging, we increased the proportion of melanocytes in the cultures to 69% and >95%, respectively, after the first and second passages (Fig. 2A). The cultures maintained >95% purity from passages 2 through 6 (data not shown). The identity of melanocytic cells in these cultures was further confirmed by their ability to oxidize L-DOPA to a dark brown pigment (Fig. 2A). Analysis of melanocytic cell markers demonstrated detectable levels of *Kit*, *Mitf-M*, *Sox10*, *Tyr* and *Trp1* transcripts in these cultures as early as passage (P)1 (Fig. 2B). The abundance of these markers increased with the purity of the cultures, and was maintained for as long as 60 days, the last time analyzed corresponding to P6 cells (Fig. 2B).

We next determined the growth characteristics of the cultured melanocytes. We observed that P2 and P3 cultures maintained normal proliferation as late as 28 days after plating, and exhibited doubling times of about 10 days (Fig. 2C). In contrast, little if any cell growth was observed in cultures at P4, P5 and P6 (Fig. 2C). Thus, under our culture conditions, primary melanocytes remain exponentially proliferative as late as 50 days following initial isolation. Significantly, whereas the average melanin content of P2 and P3 cultures was 54.3 and 66.7 µg/10^6^ cells, respectively, it increased almost threefold in P4 and P6 cultures, coinciding with pronounced morphological changes characterized by reduced nucleus:cytoplasm surface ratios and increased size, changes typical of senescent cells (Fig. 2D; Fig. S1). Mouse melanocytes escape senescence through inactivation of *Cdkn2a*, which encodes the cyclin-dependent kinase inhibitor p16^Ink4a^. Significantly, the reduction in proliferative capacity and development of senescence we observed in higher passage melanocytes was not accompanied by increases in *Cdkn2a* transcripts. Based on the observed growth characteristics of these cells, all subsequent experiments were conducted with >95% pure P2 or P3 cultures.

### Effect of extracellular matrix substrates on mouse melanocyte dendricity

**Fig. 3.**
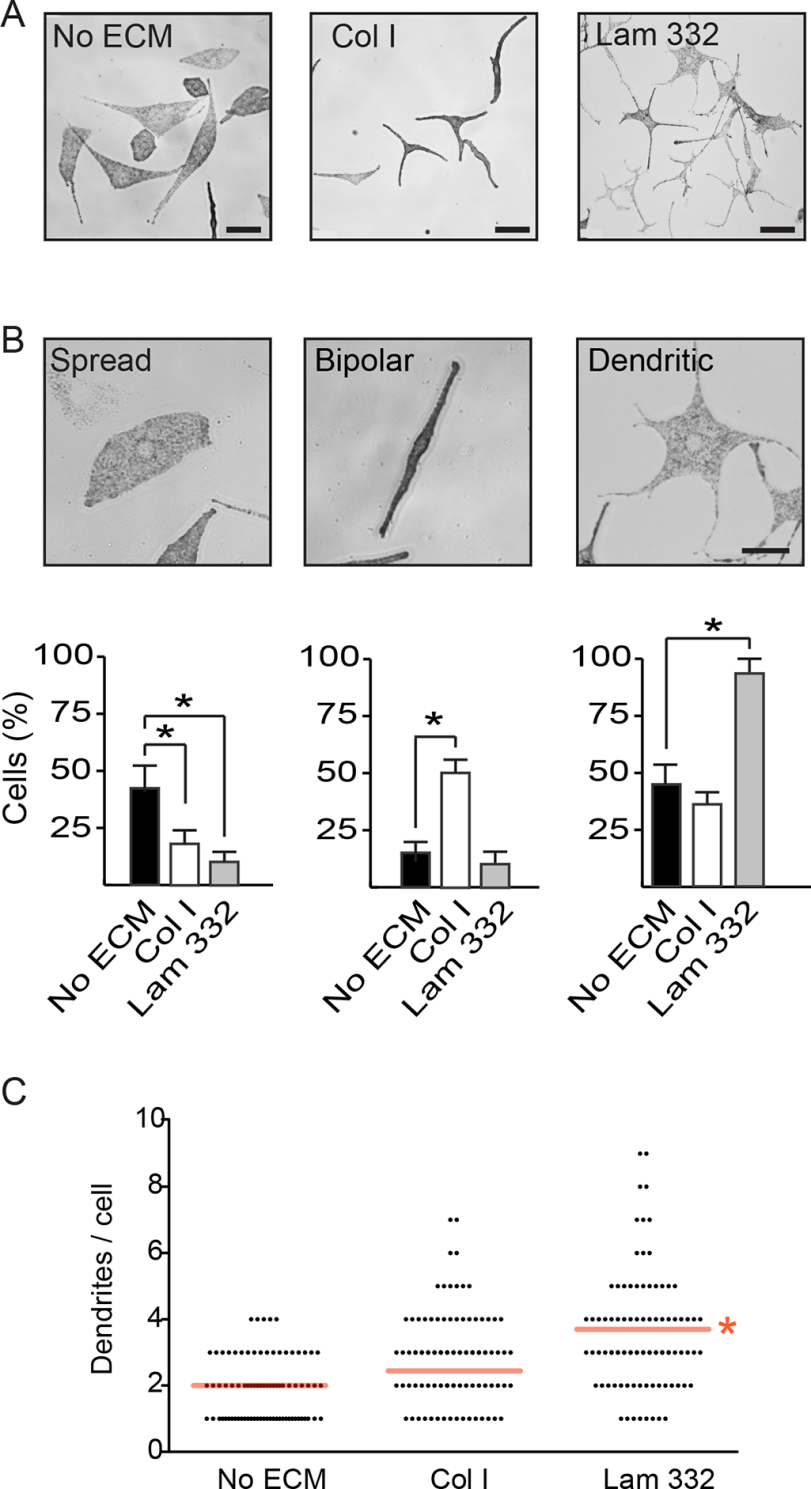
**Laminin-332 induces dendricity in primary mouse melanocytes.** (A) Phase-contrast micrographs of melanocytes cultured 24 h in the absence of exogenous extracellular matrix substrates (No ECM), on collagen I (Col I; 15 µg/cm^2^) or laminin-332 substrate (Lam 332). Scale bar: 64 µm. (B) The fraction of melanocytes exhibiting a ‘Spread’, ‘Bipolar’ or ‘Dendritic’ morphology was assessed on cultures seeded without exogenous extracellular matrix, on collagen I or on laminin-332. Each experiment was conducted in duplicate samples, and at least 50 cells per sample were scored. The results are expressed as the mean+s.d. (*n*=3 independent cell isolates). *P<0.05 (one-way ANOVA). Scale bar: 25 µm. (C) Distribution of dendrite abundance in melanocytes seeded on the indicated substrates. Melanocytes without dendrites (herewith defined as processes ≥13 µm) have been excluded. Red lines indicate the mean number of dendrites/cell on each substrate, irrespective of whether or not dendrites exhibited branches. Branches on dendrites were not counted. *P<0.05 (*n*=150 cells from 3 independent isolates; one-way ANOVA).

Melanocytes *in situ* interact with extracellular matrix (ECM) proteins in the skin basement membrane through proteins that include integrins α3β1, α6β1 and αvβ1. In culture, human primary melanocytes additionally express integrins α5β1 and αvβ3 (Hakkinen et al., 2001), which allow them to interact with substrates such as fibronectin, collagen and laminins. In embryonic and in postnatal intact epidermis, melanocytes interact with a basement membrane mainly composed of laminin-332. On the other hand, melanocytes, keratinocytes and fibroblasts in wounded epidermis play important roles in wound healing and subsequent scar formation. In particular, melanocytes can mitigate keratinocyte-dependent contraction in co-culture models of wounds composed of fibroblasts, keratinocytes and melanocytes in the presence of collagen I. Melanocytes may thus contribute to reducing the formation of fibrotic scars through interactions with collagen I, a major ECM substrate found in wounded skin (Rakar et al., 2015). In an effort to better understand melanocyte responses to ECM substrates, we next addressed whether melanocytes exhibit different responses when stimulated by laminin-332 or collagen I. Because laminin-binding integrins can mediate cellular responses distinct from those elicited by fibronectin- or collagen-binding integrins, we first compared the morphology and ability of primary mouse melanocytes to migrate on no exogenous ECM, low or intermediate concentrations of collagen I (5 µg/cm^2^ or 15 µg/cm^2^), or laminin-332 isolated from 804G rat bladder epithelial cells. Cells cultured in the absence of exogenous ECM were able to spread, frequently exhibiting polygonal morphologies with or without formation of dendrites (Fig. 3A). In contrast, melanocytes seeded on collagen adopted an elongated, and frequently bipolar, morphology (Fig. 3A and data not shown). The presence of laminin-332 induced formation of extensive and frequently branched dendritic extensions (Fig. 3A). Quantification of the fraction of cells that exhibited a spread, bipolar or dendritic phenotype revealed that in the absence of exogenous matrices about 40% of cells were either spread or dendritic and in the presence of 15 µg/cm^2^ collagen I, about 50% of the cells were bipolar, whereas 87% of melanocytes seeded on laminin-332 were dendritic (Fig. 3B). Of note, laminin-332 also increased significantly the average number of dendrites per cell. Specifically, only 6% and 25% of cells seeded without exogenous ECM or on collagen, respectively, showed >3 dendrites/cell, whereas almost 45% of cells in contact with laminin-332 formed >3 dendritic extensions (Fig. 3C). Thus, primary mouse melanocytes exhibit differential responses to various ECM substrates, and their interaction with laminin-332 appears to promote the formation of dendritic processes. The optimal formation of dendrites is essential for normal interactions between melanocytes and adjacent keratinocytes in the epidermis.

### Effect of ECM substrates on melanocyte motility

**Fig. 4.**
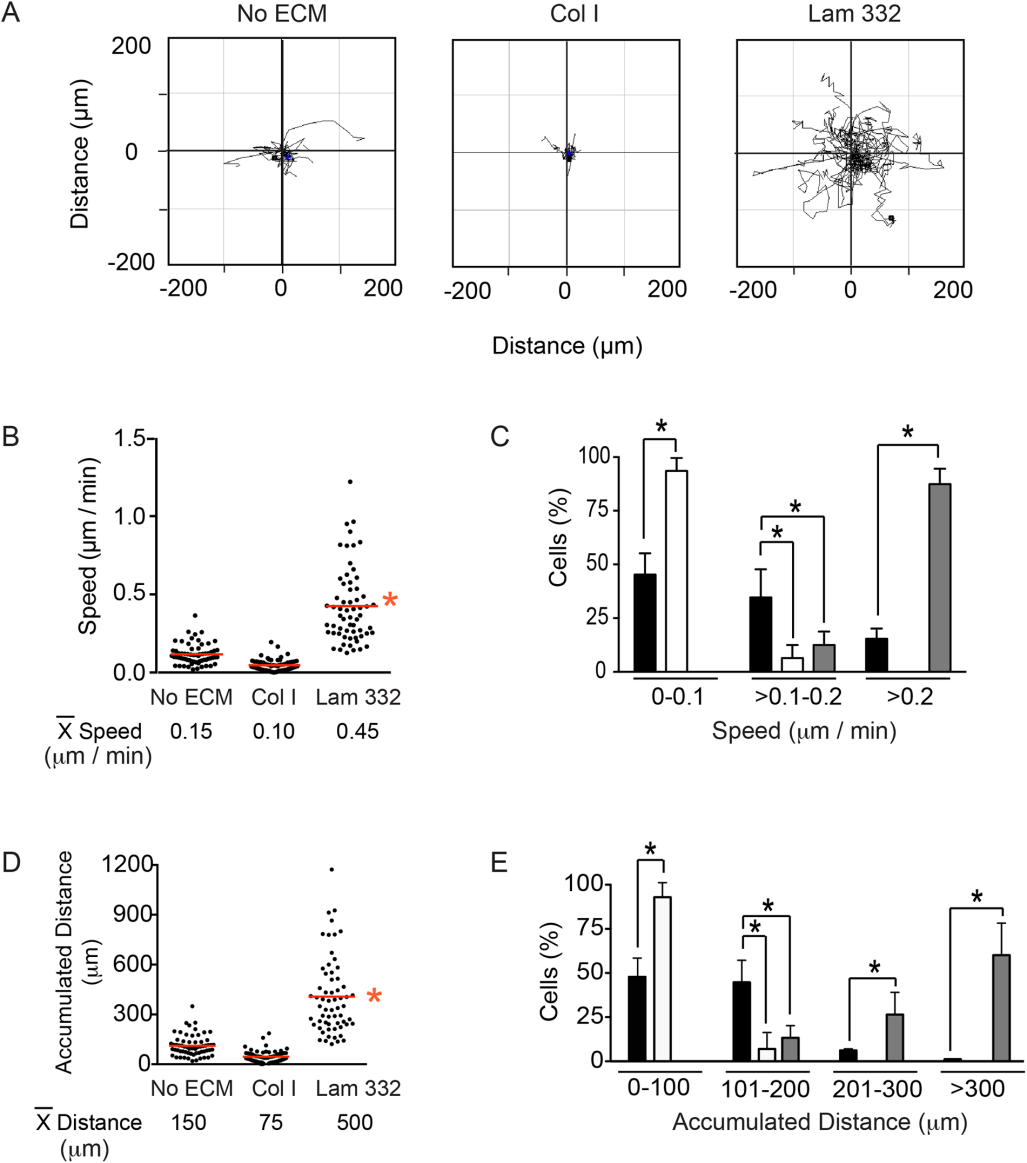
**Laminin-332 promotes melanocyte motility.** (A) Spider plots for random migration tracks of melanocytes plated without exogenous extracellular matrix (No ECM), on collagen I (Col I) or laminin-332 substrate (Lam 332), and analyzed for 16 h. (B) The particle analysis function of ImageJ was used to examine the speed of individual melanocytes on the indicated extracellular matrix substrates, with the mean speed indicated by red lines. (C) Percentage of cells that exhibited speed values within the indicated ranges when seeded on no exogenous matrix (black bars), on collagen I (white bars) or on laminin-332 (gray bars). (D) The ‘Chemotaxis and Migration Tool’ plugin for ImageJ (Ibidi) was used to examine the accumulated distance of individual melanocytes on the indicated extracellular matrix substrates, with the mean distance indicated by red lines. (E) Percentage of cells that exhibited accumulated distance values within the indicated ranges when seeded on no exogenous ECM (black bars), on collagen I (white bars) or on laminin-332 (gray bars). The data shown in the histograms (C,E) represent the mean+s.d. (*n*=300 cells analyzed from three independent isolates, 100 cells scored per isolate, one-way ANOVA). *P<0.05 for B-E.

In addition to regulating morphology, integrins enable melanocytes to interact with and migrate on ECM substrates (Pinon and Wehrle-Haller, 2011). To assess the effect of collagen and laminin on motility, we seeded melanocytes at single-cell densities and followed their movement for 16 h using time-lapse video microscopy. Plots of cell trajectories revealed substantial differences in melanocyte motility, depending on the underlying substrate. Cells seeded in the absence of exogenous ECM or on collagen I (15 µg/cm^2^) displayed average speeds of 0.12 µm/min and 0.05 µm/min, respectively. The presence of laminin-332 increased cell speed four- to ninefold, to 0.47 µm/min (Fig. 4A,B). Over 75% of cells plated without exogenous ECM or on collagen I exhibited speeds lower than 0.2 µm/min. In stark contrast, 90% of cells seeded on laminin-332 showed speeds that exceeded 0.2 µm/min (Fig. 4C). In agreement with the stimulatory effects of laminin-332 on melanocyte speed, we also observed an increase in mean accumulated distance migrated during the course of this experiment to about 450 µm, compared with 48 µm for cells plated on collagen, and 112 µm in cells plated without exogenous ECM, representing a fivefold increase (Fig. 4D). Further analysis revealed that in the presence of collagen, up to 85% of cells did not migrate distances greater than 100 µm, whereas laminin-332 promoted migration distances greater than 200 µm in 85% of melanocytes (Fig. 4E). We conclude that, in the concentrations used in these studies, laminin-332 promotes, whereas collagen appears to hamper, random melanocyte motility.

**Fig. 5.**
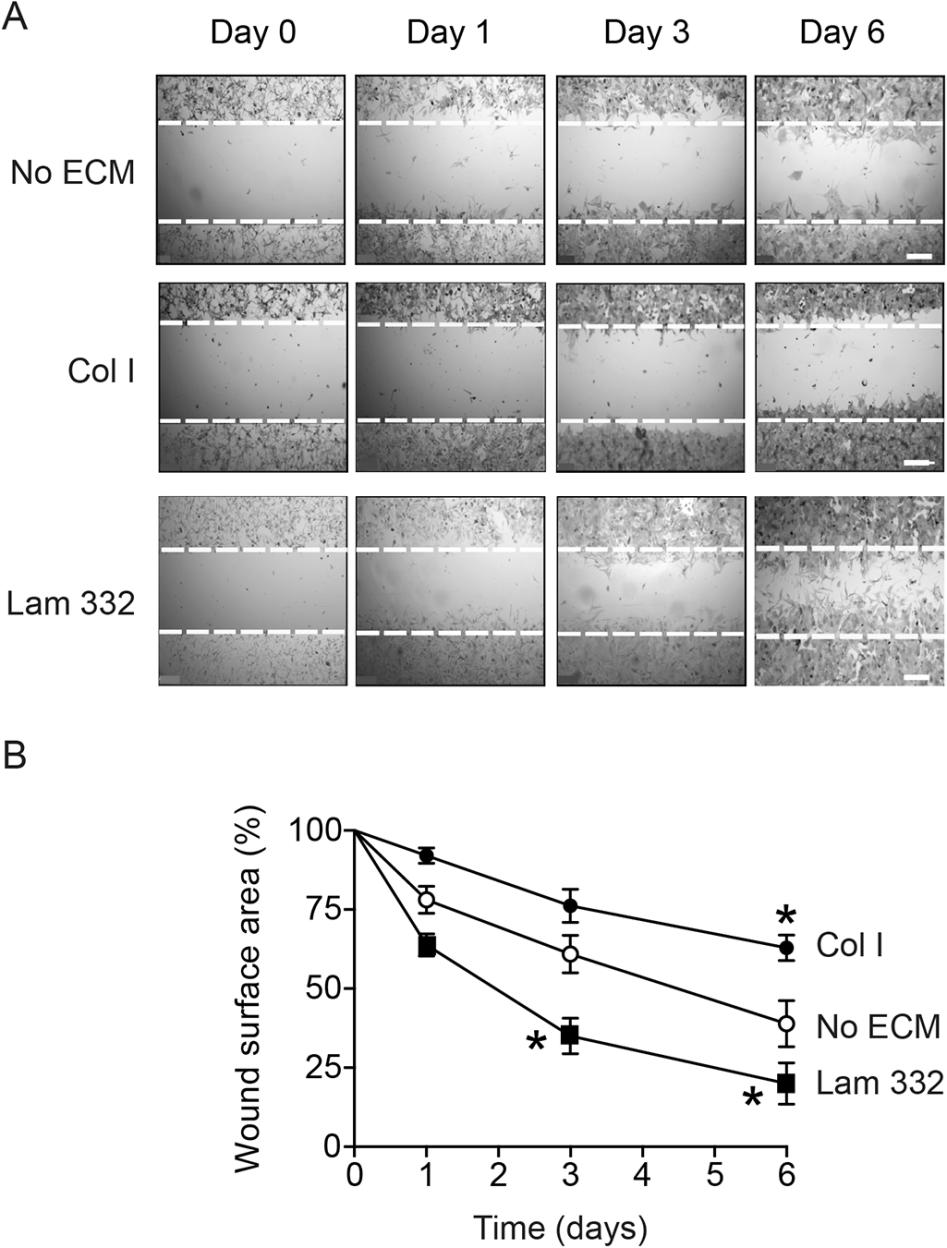
**Laminin-332 promotes directional melanocyte migration.** (A) Phase-contrast micrographs of melanocytes cultured in the absence of exogenous extracellular matrix substrates (No ECM), on collagen I (Col I) or laminin-332 substrate (Lam 332). Confluent cultures were wound-scraped, and phase-contrast images were obtained at the indicated times following wounding. The broken lines represent the width of the scrape-wound at time=0. Scale bars: 250 µm. (B) Cell-free surface areas of scrape-wounds remaining at the indicated times post-wounding were assessed using ImageJ. The results show the mean wound surface area values±s.d. (*n*=5 different cell isolates; two technical replicates per cell isolate), and have been normalized to the original scrape-wound area, which is set to 100%. *P<0.05 relative to cells plated without exogenous ECM at the corresponding time following wounding (two-way ANOVA).

The distinct effects of collagen and laminin-332 on random melanocyte motility prompted us to investigate their effects on directional migration, using scrape-wound assays. We evaluated the cell-free surface area remaining at timed intervals after wounding. Within one and three days after wounding, cells plated on laminin-332 had covered 40, 65 and 80% of the wound area in 1, 3 and 6 days, respectively (Fig. 5). By comparison, those melanocytes without exogenous ECM had covered 22, 40 and 60% of the original denuded area 1, 3 and 6 days after wounding (Fig. 5). In stark contrast, cells plated on collagen I (15 µg/cm^2^) had barely covered about 35% of the cell-free area as late as 6 days following wounding (Fig. 5). Together our data show that laminin-332, but not collagen I, induces random motility and directional migration in primary mouse melanocytes.

## DISCUSSION

Through the combined use of a transgenic mouse strain that specifically expresses Cre recombinase in cutaneous melanocytic lineage cells (Bosenberg et al., 2006), together with the ROSA^mT/mG^ double fluorescent protein reporter background, we were able to efficiently trace Cre-targeted embryonic melanocytes both in interfollicular epidermis and in the developing hair follicles. The presence of mGFP allows the simultaneous analyses by immunofluorescence microscopy of multiple proteins within a clearly identified targeted melanocytic cell, an approach that has remained difficult with various other melanocytic reporter mouse models. Following *in utero* induction at E11.5, the fraction of mGFP-positive cells observed in E20.5 embryos constituted about 10-30% of DCT-positive melanocytes, in line with the targeting efficiency reported for the original *Tyr::CreER^T2^* transgenic mouse line (Bosenberg et al., 2006).

Melanocytes derive from multipotent neural crest cells, after they delaminate from the dorsal neuroepithelium, and are thought to arise from two different developmental waves (Baggiolini et al., 2015; Mort et al., 2015). The first wave of cutaneous melanocytes arises directly from neural crest cell-derived progenitors that first migrate dorsolaterally, and then ventrally through the dermis to finally populate the epidermis and hair follicles (Adameyko et al., 2009). In these cells, the genes encoding tyrosinase, TRP-1 and dopachrome tautomerase, three major enzymes involved in the synthesis of melanin, are expressed shortly after E9 (Steingrimsson et al., 2004). A second subset of melanocytes arises later, from neural crest-derived Schwann cell progenitors found along the ventral pathway (Nitzan et al., 2013), which express MITF, TYR, and TRP-1 later than those directly derived from neural crest cells (Adameyko et al., 2009; Mort et al., 2015). Administration of tamoxifen at E10-11.5 to our reporter mice allows selective labeling of the first wave of migrating melanoblasts, excluding those generated later from Schwan cell precursors.

In culture, we have observed that 4-hydroxytamoxifen treatment results in activation of mGFP expression in ≥80% of melanocytes (data not shown), whereas no mGFP-positive cells are observed in untreated or vehicle-treated cultures. Our model thus demonstrates tight spatial and temporal regulation of genetic recombination in melanocytic cells. The generation of pure primary melanocyte cultures is challenging, as they constitute only 3-7% of all epidermal cells (Hsu et al., 2005). Several reports describing a variety of growth conditions for human and mouse melanocytes exist, including the use of phorbol ester and cholera toxin, which yield cultures with doubling times of about 5 and 12 days, respectively (Eisinger and Marko, 1982). Neonatal mouse melanocytes have been successfully cultured in medium containing placental extracts (Larue et al., 1992), or in the presence of keratinocyte feeder layers (Godwin et al., 2014). For our studies, we used melanocyte growth medium supplemented with ET-3, which was found to improve plating efficiency and cell growth (data not shown).

Under our culture conditions, mouse melanocytes in a C57/BL6 strain background retain full proliferative capacity for up to 7-9 weeks following isolation, with a doubling time of about 10 days. Although our studies did not measure the number of cell doublings that occurred in mixed melanocyte-keratinocyte cultures prior to P2, we estimate that the primary melanocyte cultures remain proliferative during approximately 9-10 population doublings, at which time they senesce. In the presence of keratinocyte feeder layers, primary mouse melanocytes reportedly exhibit doubling times of about 4-5 days, and become senescent after 4-5 weeks in culture (Sviderskaya et al., 2002). Notably, immortalization of mouse melanocytes through spontaneous or targeted inactivation of the *Cdkn2a* locus yields cells with doubling times as low as 2.3 days, which, however, also display substantially reduced melanogenic capacity (Sviderskaya et al., 2002), potentially as a result of increased genetic instability (Fung et al., 2013). Thus, our isolation and culture conditions allow for expansion and characterization of mouse melanocytes without the need of feeder layers and, because they retain full capacity to produce melanin, they are ideal to investigate the consequences of targeted genetic manipulations on melanogenic functions.

In intact epidermis, melanocyte adhesion to the basement membrane involves binding of α6β1 integrins to laminin-332 secreted by neighboring epidermal keratinocytes, as well as interactions with collagen IV and collagen XVII (Hara et al., 1994; Tanimura et al., 2011). In culture, laminin-111 and collagen IV also promote formation of dendrites in human melanocytes, whereas fibronectin or vitronectin induce bipolar or polygonal morphologies. We now show that mouse melanocytes exhibit substantially different responses to laminin-332 and collagen I, developing numerous dendrites in the presence of the former, but not the latter ECM substrate. Further, the characterization and interpretation of primary cultured melanocyte responses on a given ECM substrate, such as laminin-332, may also be critically dependent on the origin of the ECM tested. Further, different maturation/processing products can induce distinct adhesive and/or migratory cellular responses (Rousselle and Beck, 2013). Laminin-332 is synthesized by epidermal keratinocytes and other epithelial cells as a 460-kDa precursor protein, which is secreted into the ECM milieu and subsequently processed into smaller forms (Rousselle and Beck, 2013), and laminin-332 from rat bladder epithelial 804G cells differs from that secreted by SCC25 squamous carcinoma cells or HaCaT immortalized human keratinocytes (Scott et al., 1999; Zhang et al., 2013). Functionally, laminin-332 from 804G cells is vastly superior in supporting adhesion and clonal growth of primary human melanocytes than HaCaT-derived laminin-332 (Scott et al., 1999; Zhang et al., 2013). Similarly, whereas we observed pronounced positive effects of 804G-derived laminin-332 on both random and directional migration in our melanocyte cultures, SCC25-derived laminin-332 matrix failed to stimulate random migration in human melanocytes (Scott et al., 1999).

Functional differences also exist between laminin- and collagen-binding integrins (Stipp, 2010). For example, responses of epithelial cells to laminins frequently involve strong activation of Rac1 and Cdc42, which induce formation of lamellipodia and filopodia, respectively, as well as formation of smaller focal contacts and a dynamic actin cytoskeleton that promotes rapid migration. In contrast, responses to collagen or fibronectin are frequently associated with RhoA activation, formation of robust stress fibers and focal adhesions (Stipp, 2010). Cell motility and adhesion are also influenced by ECM substrate concentration. For example, high concentrations of fibronectin or collagen (>30 µg/cm^2^) promote cell adhesion and spreading, and inhibit formation of cell protrusions and forward movement. Conversely, intermediate concentrations (5-10 µg/cm^2^) provide optimal levels of cell-matrix adhesion and ability to form protrusions, which facilitate migration (Cox et al., 2001; Gehler et al., 2017). Although melanocytes are not epithelial in origin, we similarly observed optimal migration on laminin-332 matrix, compared to intermediate concentrations of collagen I. However, unlike epithelial and mesenchymal cells, primary melanocytes did not display a spread morphology when cultured on collagen I, but rather adopted a long bipolar morphology with reduced dendrite formation, and occupied smaller surface areas than those seeded on laminin or without exogenous ECM. Primary melanocytes are known to shed melanosomes primarily through dendritic extensions (Wu and Hammer, 2014), although shedding events can also be induced from central areas in cells seeded on collagen-rich substrates (Wu et al., 2012). Given the effects of various extracellular matrices on melanocyte morphology and dendricity, an important area of future research will be to systematically examine the effect of ECM substrates on the diverse pathways that modulate melanosome shedding by melanocytes and their uptake by neighboring epidermal keratinocytes.

In conclusion, we have developed a mouse model for *in vivo* tracing of melanocytic lineage cells. We have also developed efficient isolation and culture methods to systematically examine the characteristics of mouse primary melanocytes and their responses to various ECM substrates. Our studies underline the importance of clearly defining culture conditions and cell passage number during characterization of melanocyte biological processes. The combined use of our mouse reporter model and our clearly defined and characterized culture systems will provide valuable tools to decipher the cellular and molecular mechanisms that regulate melanocytic cell behavior, and how their alterations may contribute to neurocristopathies and pigmentation disorders.

## MATERIALS AND METHODS

### Mouse strains

All animal experiments were approved by the University of Western Ontario Animal Care Committee (Protocol No. 2016-022), in accordance with regulations and guidelines from the Canadian Council on Animal Care. The mice used for these studies express Cre recombinase fused to a modified estrogen receptor under the control of the *Tyr* promoter (Bosenberg et al., 2006). For *in vivo* lineage tracing studies, these mice were bred to homozygosity with mice containing ROSA^mT/mG^ Cre reporter allele background (Muzumdar et al., 2007). Genotyping was conducted as previously described (Sayedyahossein et al., 2015). To induce Cre recombinase activation, tamoxifen (1 mg/25 g body weight dissolved in ethanol:Cremophore:PBS 1:1:5 v/v) was administered intraperitoneally once to 11.5 days post-coitus (dpc) pregnant dams. 0.5 dpc for timed pregnancies was considered to be at midday of the day vaginal plugs appeared. *In vivo* tracing studies were conducted with 3-5 embryos from at least two litters.

### Reagents and antibodies

Rat tail Collagen Type I (354236) was from Corning Inc. (NY, USA), endothelin-3 (CC-4510) was purchased from Lonza (Walkersville, MD, USA), Permount mounting medium (SP15-100) was from Fisher Scientific (Ottawa, Ontario, Canada). Cremophor (Kolliphor EL; C-5135), L-DOPA (59-92-7), synthetic melanin (M8631) and all other chemicals were purchased from Sigma (St. Louis, MO, USA). Antibodies purchased from Abcam (Cambridge, UK) were: Mouse anti-TRP1 [cat. no. 3312, lot no. GR239310-1, diluted 1:200 in phosphate-buffered saline (PBS) containing 5% goat serum] (Delevoye et al., 2009) and chicken anti-GFP (cat. no. 13970, lot no. GR279236-1, diluted 1:1000 in PBS containing 5% goat serum) (Jackson et al., 2015). The rabbit anti-keratin 14 antibody (cat. no. CLPRB-155P, lot no. D14IF01918, diluted 1:1000 in PBS containing 5% goat serum) (Jackson et al., 2015) was from Covance (Cranford, NJ, USA). The goat polyclonal anti-DCT antibody (cat. no. 10452, lot no. 10616, diluted 1:100 in PBS containing 5% donkey serum) (Smith et al., 2013) was purchased from Santa Cruz Biotechnology (CA, USA). Alexa Fluor^®^-conjugated goat anti-mouse and goat anti-rabbit IgG were purchased from Molecular Probes/Invitrogen (Eugene, OR, USA).

### Primary melanocyte culture and proliferation measurements

Melanocytes from all experiments were isolated from animals not exposed to tamoxifen. Skin was harvested from 3-day-old mice of both sexes and digested with Dispase II (5 U/ml, 4942078001, Roche, Switzerland) for 1.5 h at 37°C. The epidermis was mechanically separated, minced and digested with a 0.025% Trypsin/ 0.01% EDTA solution (R-001-100, Thermo Fisher, Carlsbad, CA, USA) for 10 min at 37°C, with gentle rocking. Two volumes of trypsin neutralizer solution (R002100, Thermo Fisher) were added, and a cell suspension was obtained by thorough mixing. Tissue debris and cornified envelope fragments were removed by filtration through a 100-µm pore size nylon strainer (08-771-19, Corning Inc.), and the resulting cell suspension was centrifuged (200×***g***, 10 min, 22°C). Pelleted cells were suspended in MBM-4 medium (CC-4435, Lonza) supplemented with endothelin-3 (260 ng/ml, final) and growth factors (CC-4435, Lonza), and seeded at a density of 1×10^5^ cells/cm^2^. The growth medium was replaced one day after plating, and subsequently every 72 h. The cultures were consistently maintained at 30-90% confluence. Where indicated, cell culture surfaces were coated with rat-tail Collagen Type I (15 µg/cm^2^) or laminin-332 matrix obtained from 804G rat bladder squamous carcinoma cell conditioned medium (Tripathi et al., 2008). To this end, confluent 804G cultures were rinsed with PBS, and maintained in serum-free Eagle's Minimum Essential Medium for 48 h. The spent medium was filtered to remove cell debris, and used to coat culture surfaces by incubation at 37°C for 1-2 h prior to plating melanocytes. For proliferation measurements, triplicate samples from independent cell isolates were seeded at a density of 15,000 cells/well in 24-well culture dishes. Cell numbers were determined weekly from trypsinized cultures. Unless otherwise indicated, all experiments were conducted with melanocytes passaged 2-3 times.

### Immunohistochemistry and immunofluorescence microscopy

Paraffin-embedded 7-µm sections of skin harvested from E20.5 embryos were deparaffinized and subjected to high-temperature antigen retrieval, with 10 mM sodium citrate (pH 6.0), followed by incubation with primary antibodies, as described (Rudkouskaya et al., 2014). For histological visualization of melanin, tissue sections were treated with Fontana-Masson stain (150669, Abcam) and counterstained with Nuclear Fast Red, following the manufacturer's instructions. For indirect immunofluorescence microscopy analyses of cultured melanocytes, cells were fixed in freshly diluted 4% paraformaldehyde (PFA), and processed as described (Ivanova and Dagnino, 2007; Sayedyahossein et al., 2015). Fluorescence and phase-contrast micrographs were obtained with a Leica DMIRBE fluorescence microscope equipped with an ORCA-ER digital camera (Hamamatsu Photonics, Hamamatsu, Japan), using Volocity 6.1.1 software (Improvision, Coventry, UK).

### Light and time-lapse video microscopy

Time-lapse video microscopy images were obtained with a Leica DMIRBE fluorescence microscope equipped with an ORCA-ER digital camera (Hamamatsu Photonics), using Volocity 6.1.1 software (Improvision). Light microscopy images of tissue sections were obtained with a Leica DM-LRE2 microscope equipped with a ProgRes C5 camera (Jenoptik Optical Systems, Jupiter, Florida, USA) and ProgRes Mac CapturePro 2.7.6 (Jenoptik) imaging software.

### Melanogenic assays

L-DOPA reaction assays were conducted as described (Tsuchiyama et al., 2013). Briefly, cultured melanocytes were fixed in freshly diluted 4% PFA at 22°C for 20 min. The cells were rinsed thrice with phosphate-buffered saline (PBS), and were then incubated with 5 mM L-Dopa diluted in PBS (pH 6.8) at 37**°**C for 4 h, protected from light. After incubation, the cells were rinsed with water and mounted with Permount.

Melanin quantification was conducted as described (Flori et al., 2011). Melanocytes (5×10^5^ cells) were trypsinized from 60-mm culture dishes. An aliquot of this cell suspension was used to determine the number of cells present, and the remainder was centrifuged (200×***g***, 10 min, 22°C). The cell pellet was suspended and lysed in an aqueous solution of 1 M NaOH containing 10% DMSO, which was incubated at 80°C for 1 h with vigorous shaking. The lysates were clarified by centrifugation (12,000×***g***, 20 min, 22°C), and the melanin content in the lysate was determined from absorbance measurements at 420 nm, interpolating the values obtained with those from a synthetic melanin standard curve. The results were normalized to cell number.

### RNA isolation and qPCR

Total RNA was isolated using RNeasy microkits (Qiagen, Louisville, KY, USA), following the manufacturer's instructions. RNA quality was determined with an Agilent 2100 Bioanalyzer (Agilent, Santa Clara, CA, USA). RNA samples (200 ng) with integrity numbers ≥8.5 were subjected to reverse transcription (SuperScript II RT, Fisher Scientific), following the manufacturer's protocol. Quantitative, real-time polymerase chain reactions (qPCR) were conducted on a CFX384 Real-Time PCR system (Bio-Rad, Hercules, CA, USA) operated by CFX Manager software (version 1.6). cDNA samples corresponding to 20 ng of RNA were amplified using PerfeCTa qPCR SuperMix (Quanta BioSciences, Gaithersburg, MD, USA) as described (Singh and Dagnino, 2016), and using the following primers (400 nM, final): *Tyrp1* forward (F) 5′-GCCTTCTTTCTCCCTTCCTTAC-3′ and reverse (R) 5′-CTGCTGGTCTCCCTACATTTC-3′; *Tyr* (F) 5′-CTCTGGGCTTAGCAGTAGGC-3′ and (R) 5′-GCAAGCTGTGGTAGTCGTCT-3′; *Kit* (F) 5′-CCTCCTGCCCTTTATCCTTTAG-3′ and (R) 5′-GACCTCCAAACCAGCTTACTT-3′, *Sox10* (F) 5′-AGCCCTCAGGACCCTATTAT-3′ and (R) 5′-GTCAGAGATGGCAGTGTAGAG-3′**,** *Cdkn2a* (F) 5′-GCTGGGTGGTCTTTGTGTA-3′ and (R) 5′TTAGCTCTGCTCTTGGGATTG-3′. The *Mitf* (F) 5′-GAAAGTAGAGGGAGGAGGACTAA-3′ and (R) 5′-GCACCTGGTAGTGACTGTATTC-3′ primers allow amplification of the melanocyte-specific transcript variant 2, which encodes MITF-M. The results were normalized to the expression of the *Rpl16* and *Rps29 genes*, which encode ribosomal protein L16 and S29, respectively (de Jonge et al., 2007). Replicate cDNA samples were amplified for 40 cycles (98°C for 10 s, and 58°C for 30 s per cycle). Primer specificity and amplicon sizes were confirmed, respectively, by analysis of melt curves conducted between 65°C and 95°C, in 0.5°C increments and agarose gel electrophoresis. Relative mRNA levels were calculated using the ΔΔCq method. RNA preparations obtained from three different cell isolates were analyzed.

### Migration assays

For single-cell motility analyses, melanocytes were seeded at 2×10^4^ cells/35-mm μ-Dish culture dish (81156; Ibidi USA, Madison WI, USA). Time-lapse video microscopy images were obtained 24 h after plating, and cells were manually tracked using the Manual Tracking plugin for ImageJ software (version 1.49v, Fiji, NIH). The velocity, accumulated and Euclidean distances of each cell were calculated using the ‘Chemotaxis and Migration Tool’ plugin for ImageJ (version 1.01). At least 20 cells/condition were tracked in individual experiments. To assess directional migration, scrape-wound assays were conducted as described (Vi et al., 2011), with minor modifications. Melanocytes were seeded onto 24-well culture dishes with no exogenous ECM, or coated laminin-332 matrix, at a density of 1×10^5^ cells/ well. Alternatively, the cells were seeded at a density of 1.5×10^5^ cells/well on Collagen Type I-coated dishes (15 µg/cm^2^). 24 h later, a scrape wound was created with a sterile 200-µl pipette tip. The cell monolayer was rinsed with PBS to remove cell debris and cultured in growth medium. Changes in gap surface area were determined from phase-contrast micrographs obtained at timed intervals following wounding, using ImageJ software.

### Statistical analysis

Data were analysed using one- or two-way ANOVA, as indicated in individual experiments, with post hoc Tukey's correction. Significance was set at *P*<0.05. Experiments with cultured melanocytes were conducted in triplicate samples, and at least three times with independent cell isolates.
